# Whole transcriptome approach to evaluate the effect of aluminium hydroxide in ovine encephalon

**DOI:** 10.1038/s41598-020-71905-y

**Published:** 2020-09-17

**Authors:** Endika Varela-Martínez, Martin Bilbao-Arribas, Naiara Abendaño, Javier Asín, Marta Pérez, Damián de Andrés, Lluís Luján, Begoña M. Jugo

**Affiliations:** 1 Department of Genetics, Physical Anthropology and Animal Physiology, Faculty of Science and Technology, University of the Basque Country (UPV/EHU), Leioa, Spain; 2 Department of Animal Pathology, University of Zaragoza, Zaragoza, Spain; 3 Institute of Agrobiotechnology (CSIC-UPNA-Gov. Navarra), Navarra, Spain

**Keywords:** Computational biology and bioinformatics, Genetics, Immunology, Molecular biology, Diseases

## Abstract

Aluminium hydroxide adjuvants are crucial for livestock and human vaccines. Few studies have analysed their effect on the central nervous system in vivo. In this work, lambs received three different treatments of parallel subcutaneous inoculations during 16 months with aluminium-containing commercial vaccines, an equivalent dose of aluminium hydroxide or mock injections. Brain samples were sequenced by RNA-seq and miRNA-seq for the expression analysis of mRNAs, long non-coding RNAs and microRNAs and three expression comparisons were made. Although few differentially expressed genes were identified, some dysregulated genes by aluminium hydroxide alone were linked to neurological functions, the lncRNA TUNA among them, or were enriched in mitochondrial energy metabolism related functions. In the same way, the miRNA expression was mainly disrupted by the adjuvant alone treatment. Some differentially expressed miRNAs had been previously linked to neurological diseases, oxidative stress and apoptosis. In brief, in this study aluminium hydroxide alone altered the transcriptome of the encephalon to a higher degree than commercial vaccines that present a milder effect. The expression changes in the animals inoculated with aluminium hydroxide suggest mitochondrial disfunction. Further research is needed to elucidate to which extent these changes could have pathological consequences.

## Introduction

Since the 1920’s, when aluminium (Al) was discovered to enhance immune response providing more effective protection^1^ vaccines have been complemented with adjuvants. Because of the effectiveness of aluminium adjuvants at enhancing humoral responses, their good tolerance without causing fever and with the longest safety record among used adjuvants^2^ aluminium salts are preferably used in both animal and human vaccines. Nevertheless, the mechanism of enhancement of immune response by adjuvants has not been thoroughly analyzed and its importance has been underestimated for a long time^3^.

The aluminium oxyhydroxide based Alhydrogel is one of the most common aluminium-based adjuvant used in clinically authorized vaccines. The potential effect of this kind of compounds on the nervous system has been tested mainly in animal models such as mouse. In CD1 mice, with a dose of 100 μg Al/kg, subcutaneously inoculated Alhydrogel adjuvant induced cognitive alterations associated with death of motor neurons and an enormous increase (350%) of reactive astrocytic cells in an inflammatory process^4^. Moreover, with a dose of 300 μg Al/kg, microglial and astroglial reactions were detected in the spinal cord of the same mice type, and altered motor and cognitive functions were observed^5^. In an immunization experiment in mice, after the inoculation of oxyhydroxide particles fluorescently labelled, an average of 15 solid aluminium particles were detected in the mice brain at 21 days postimmunization. In vitro studies performed in parallel confirmed the toxicity of aluminium adjuvant to neuronal cell cultures^6^.

Very few studies have analysed the Al effect in animal nervous system by RNA-seq technology. In a recent work, Xu et al.^7^ identified by means of RNA-seq 96 upregulated and 652 downregulated mRNAs, and 37 dysregulated long non-coding RNAs (lncRNAs) in the hippocampus of Al treated rats. The main functions of dysregulated genes, revealed by Gene Ontology analysis, were related with glial cell differentiation, neural transmission and vesicle trafficking. Moreover, the results of this study suggested that glial cell-related genes had relevant effects in the mechanisms associated with Al neurotoxicity and that aberrant mRNAs and lncRNAs were involved in the response to Al in the analysed tissue.

Our group has characterized the effect of Al hydroxide adjuvant and its influence on the immune response to vaccination in a long term experimental design, using sheep as a model, based in total RNA and microRNAs sequencing in peripheral blood mononuclear cells (PBMCs)^8^. With the main objective of deciphering the molecular signature activated, two different treatments were applied to lambs: commercial vaccines including Al hydroxide or Alhydrogel (aluminium hydroxide gel suspension) only in an equivalent dose. In animals of both treatments the NF-kB signalling pathway was enriched, and at the end of the experiment a downregulation of cytokines and cytokine receptors was detected in the adjuvant inoculated animals in relation to the vaccinated animals. In the adjuvanted group, differential expression of six miRNAs was also detected. Thus, aluminium could induce endogenous danger signals with an effect in the stimulation of the immune system.

Long non-coding RNAs are non-coding RNAs longer than 200 nucleotides and often transcribed. They usually do not code for proteins but their spatiotemporal-specific expression patterns indicate their diversity in functions and complexity in mechanisms^9^. They are implicated in neural function and maintenance, and many neurodegenerative diseases such as Alzheimer’s disease (AD) have been linked with aberrant lncRNAs^10^. They have been also associated with chemical carcinogenicity and metal toxicity, and the relationship of some lncRNA and cadmium for example, has been reported^11^.

Thus, the main objective of this study was to identify the molecular signatures activated by vaccines and adjuvants in the form of Al hydroxide in sheep encephalon, in the same group of animals as our previous work, by combining the molecular information provided by RNA sequencing of mRNAs, miRNAs as well as lncRNAs. Moreover, the interaction between them was analysed.

## Results

### Statistics for RNA-seq data

The sequenced 12 RNA-seq libraries had an average depth of 74.1 million paired-end reads. After adaptor and quality filtering, a mean of 68.8 million reads (92.80%) remained for subsequent analyses. Those reads were aligned against the *Ovis aries* reference genome (Oar3.1), achieving the following results in average: 60.7 million read pairs (88.33%) mapped uniquely to the reference, 5.9 million read pairs (8.54%) mapped to multiple loci and 2.1 million read pairs (3.13%) not mapped to any loci. Only uniquely mapped reads were used for subsequent analyses.

### Identification and classification of lncRNAs

**Figure 1:**
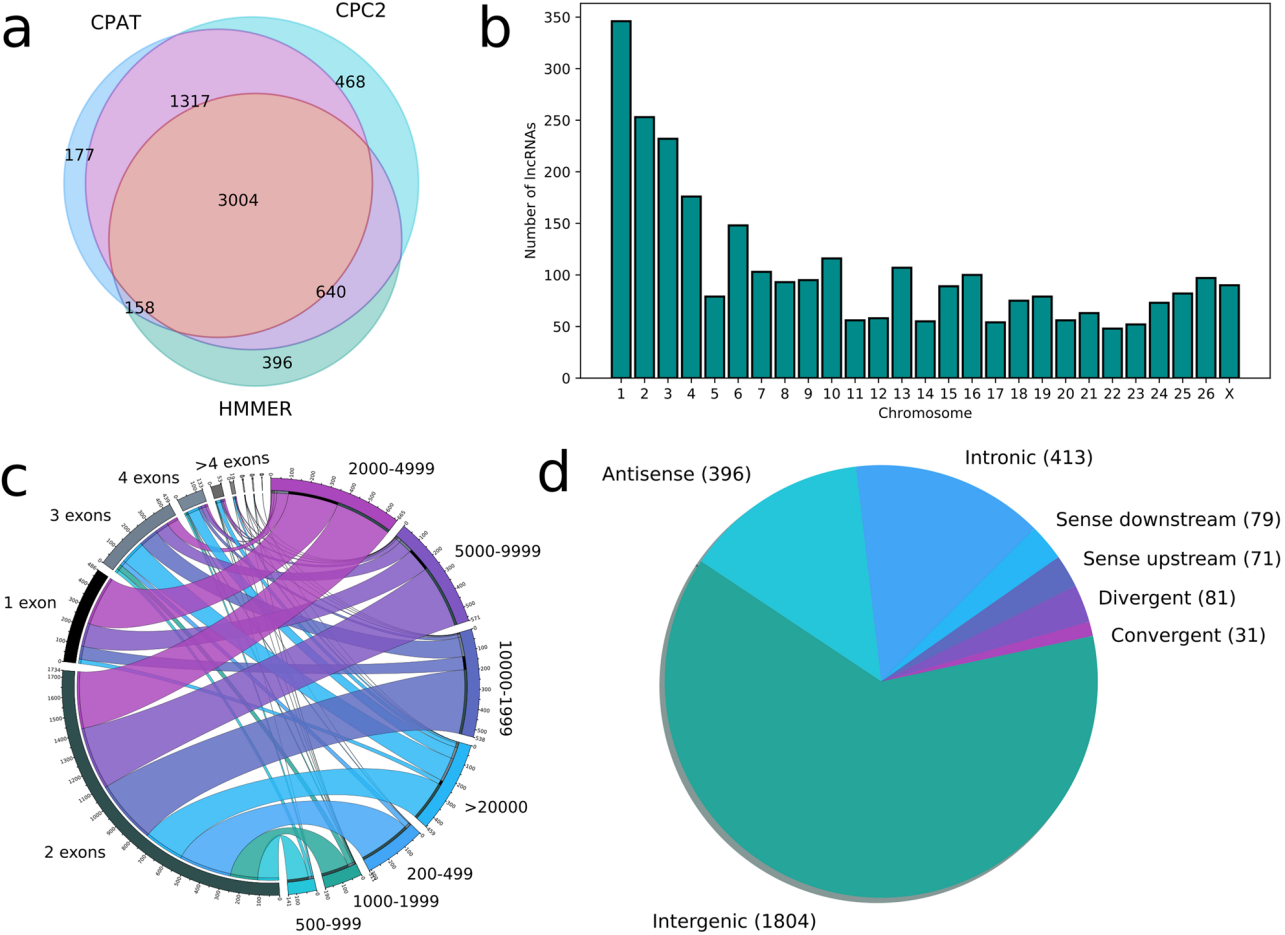
Summary statistics of the lncRNAs. (**a**) Venn diagram with the coding-potential assessment results obtained with CPAT, CPC2 and HMMER. (**b**) Distribution of lncRNA transcripts through chromosomes. (**c**) Relationship between length and exon number in the detected lncRNAs. (**d**) Classification of detected candidate lncRNAs by relative location to the closest annotated gene.

**Figure 2:**
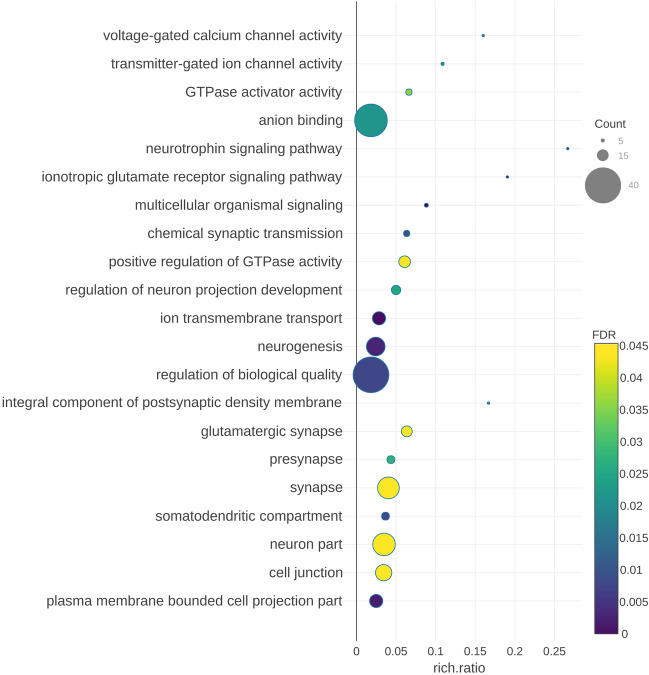
Pathway analysis of genes that harboured intronic lncRNAs. The bubble plot shows in the Y-axis the enriched pathways, while in the X-axis the rich ratio is represented (rich ratio = amount of genes in the term/total amount of genes in the enriched term). Size and colour of the bubble represents the number of genes in the GO term and enrichment significance (FDR), respectively.

Filtering steps to improve the reliability of unknown intergenic, intronic and antisense transcripts as lncRNAs reduced the list of potential lncRNAs to 3,004. Despite their different approaches, the three methods for detecting coding sequences performed in concordance, with CPAT and CPC2 giving more similar results (Fig. 1a). They are evenly distributed across all the chromosomes except for the X chromosome that harbours less transcripts than expected for its length (Fig. 1b). More than half of the transcripts are longer than 5,000 nucleotides, many of the single-exon transcripts are between 2,000 and 4,999 nucleotides long and there are few transcripts with more than 3 exons (Fig. 1c). We classified all the transcripts into different categories based on their relative location to their closest genes. Transcripts overlapping and in the same strand as known coding genes were not considered. Most lncRNAs are located in intergenic regions and those less than 5 kb apart from their neighbours are classified in their own category due to potential regulatory relations (Fig. 1d). Intronic lncRNAs showed better correlations, in average, with their closest genes than other categories and the genes that harboured these transcripts were enriched in several functions and pathways related to neuron activity (Fig. 2), while other lncRNA types did not show any overrepresented ontology or pathway terms.

In relation to the conservation of the detected lncRNAs in sheep, we identified few lncRNAs already annotated in other species through Blast searches against RNAcentral; among them, the lncRNA TUNA was detected, which was differentially expressed between the adjuvant group and the other two groups. This lncRNA has been found conserved in many vertebrates like cattle (URS00008E3A0F) or human (URS000075CAB8). We also identified, albeit with incomplete alignments, similar transcripts to other human lncRNAs such as *NORAD*, *HCG11* or *COPG2IT1*.

### Analysis of differential expression of mRNAs and lncRNAs

First, lowly expressed genes, defined as those with an expression lower than 1 CPM and found in less than four individual libraries, were filtered out from the differential expression analysis. Thus, 16,369 genes remained for subsequent analysis, of which 14,387 were annotated genes in Ensembl and 1,982 were candidate lncRNAs. One sample from the adjuvant group was treated as an outlier and was extracted from the analysis.

**Figure 3:**
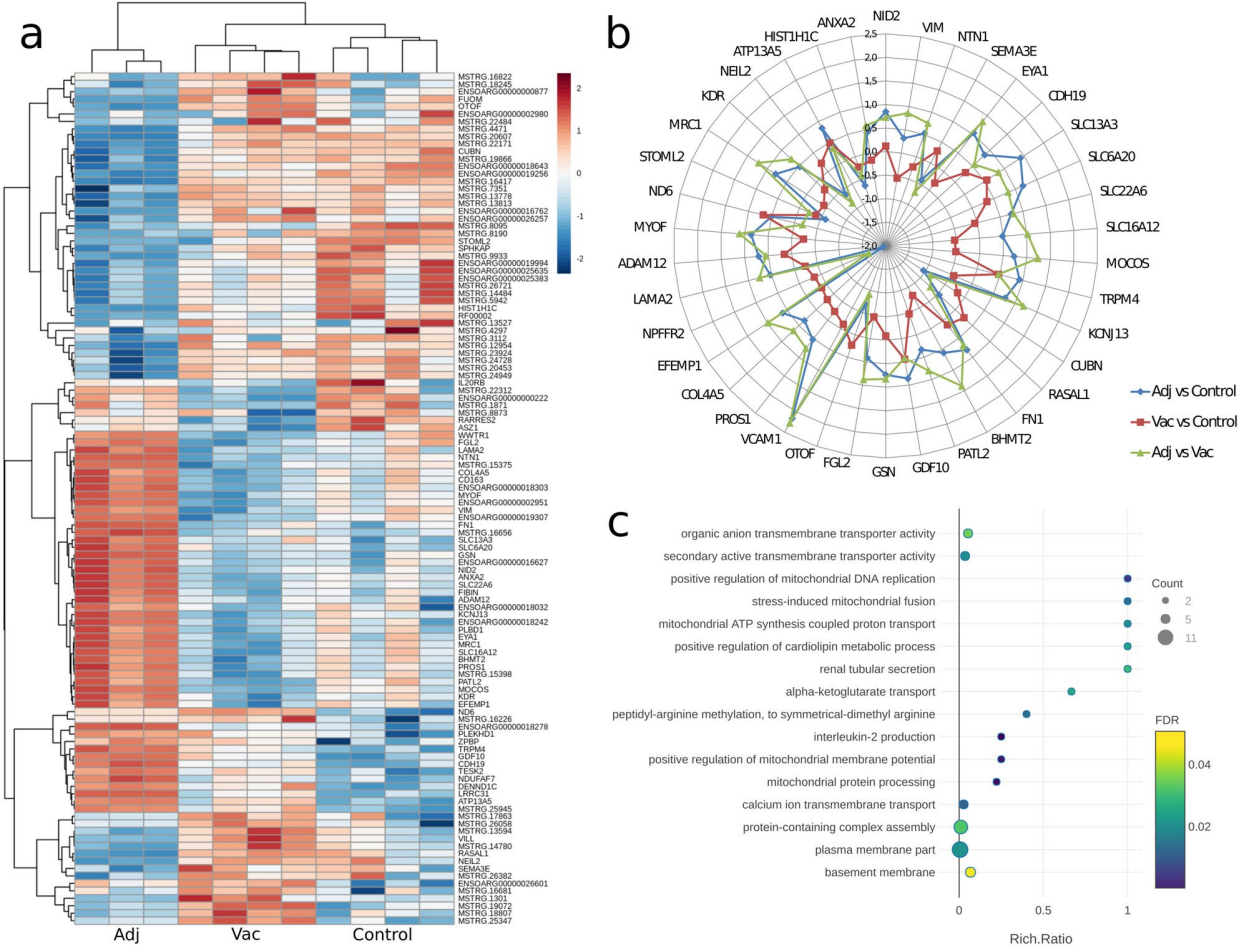
Differential expression of coding and lncRNA genes. (**a**) Heatmap depicting all the differentially expressed genes in Adj vs. Control, Vac vs. Control and Adj vs.Vac comparisons. (**b**) Radar plot with the log_2_FC of overrepresented genes related to neuronal development, neurotransmission and neurodegenerative diseases in Adj vs. Control (blue), Vac vs. Control (red) and Adj vs.Vac (green) comparisons. (**c**) GO enrichment term analysis of differentially expressed genes in the Adj vs. Control and Adj vs. Vac comparisons. The bubble plot shows in the Y-axis the enriched GO terms, while in the X-axis the rich ratio is represented (rich ratio = amount of differentially expressed genes in the term/all genes included in the term). Size and colour of the bubble represent the number of differentially expressed genes in the GO term and enrichment significance (FDR), respectively.

In the Adj vs. Control comparison 63 DEGs were identified, including 33 genes, of which 20 were up-regulated and 13 were down-regulated, and 30 new lncRNAs consisting of 3 that were up-regulated and 27 down-regulated. In the Vac vs. Control comparison 13 DEGs were identified, including 6 genes, of which 2 were up-regulated and 4 were down-regulated, and 7 new lncRNAs consisting of 5 that were up-regulated and 2 down-regulated. Furthermore, in the Adj vs. Vac comparison 76 DEGs were identified, including 45 genes, of which 33 were up-regulated and 12 were down-regulated, and 31 new lncRNAs consisting of 4 that were up-regulated and 27 down-regulated. A detailed list of the DEGs can be seen as a heatmap (Fig. 3a), while in Supplementary Dataset S1 it can be seen a detailed summary of the differential expression analysis for all genes that passed the filtering criteria and differentially expressed lncRNAs in Supplementary Dataset S2.

Within the DE-mRNAs are factors that are clearly related to neuronal development (*NID2, VIM, NTN1, SEMA3, EYA1, CDH19*), brain transport and neurotransmission (*SLC13A3, SLC6A20, SLC6A12, MOCOS, TRPM4, KCNJ13, CUBN, MRASAL1*), brain injury (*FN1, BHMT2, PATL2, GDF10, GSN, FGL2, OTOF, VCAM1, PROS1, COL4A5, EFEMP1, NPFFR2, LAMA2, ADAM12, MYOF*) and neurodegenerative diseases associated with Al like AD (*ND6, STOML2, MRC1, KDR, NEIL2*), Parkinson Disease (PD) (*ATP13A5, HIST1H1C*) and Amyotrophic Lateral Sclerosis (ALS) (*ANXA2*) (Fig. 3b).

### Validation of RNA-seq data

To validate RNA-seq data, 13 mRNAs were verified by RT-qPCR. Fold changes in expression between the different groups are shown in Supplementary Fig. S2. Data from RNA-seq and RT-qPCR showed a high degree of concordance and there were no significant differences in fold change data obtained with RNA-seq and RT-qPCR (p > 0.05), indicating that the sequence results are reliable.

### Functional annotation and classification for RNA-seq data

Functional characterization of the DE-mRNAs was performed with PANTHER to identify enriched GO terms in the three domains: Cellular Component (CC), Molecular Function (MF) and Biological Process (BP). In the Adj vs. Control comparison, 27 significantly overrepresented GO terms (with an adjusted p-value < 0.05) were identified in total. Among the top ranked Biological Processes were positive regulation of mitochondrial DNA replication (GO:0090297), stress-induced mitochondrial fusion (GO:1990046), mitochondrial ATP synthesis coupled proton transport (GO:0042776), positive regulation of cardiolipin metabolic process (GO:1900210), alpha-ketoglutarate transport (GO:0015742), peptidyl-arginine methylation to symmetrical-dimethyl arginine (GO:0019918), positive regulation of mitochondrial membrane potential (GO:0010918), mitochondrial protein processing (GO:0034982) and calcium ion transmembrane transport (GO:0070588) (Fig. 3c).

### Results from the weighted gene co-expression network analysis

**Figure 4:**
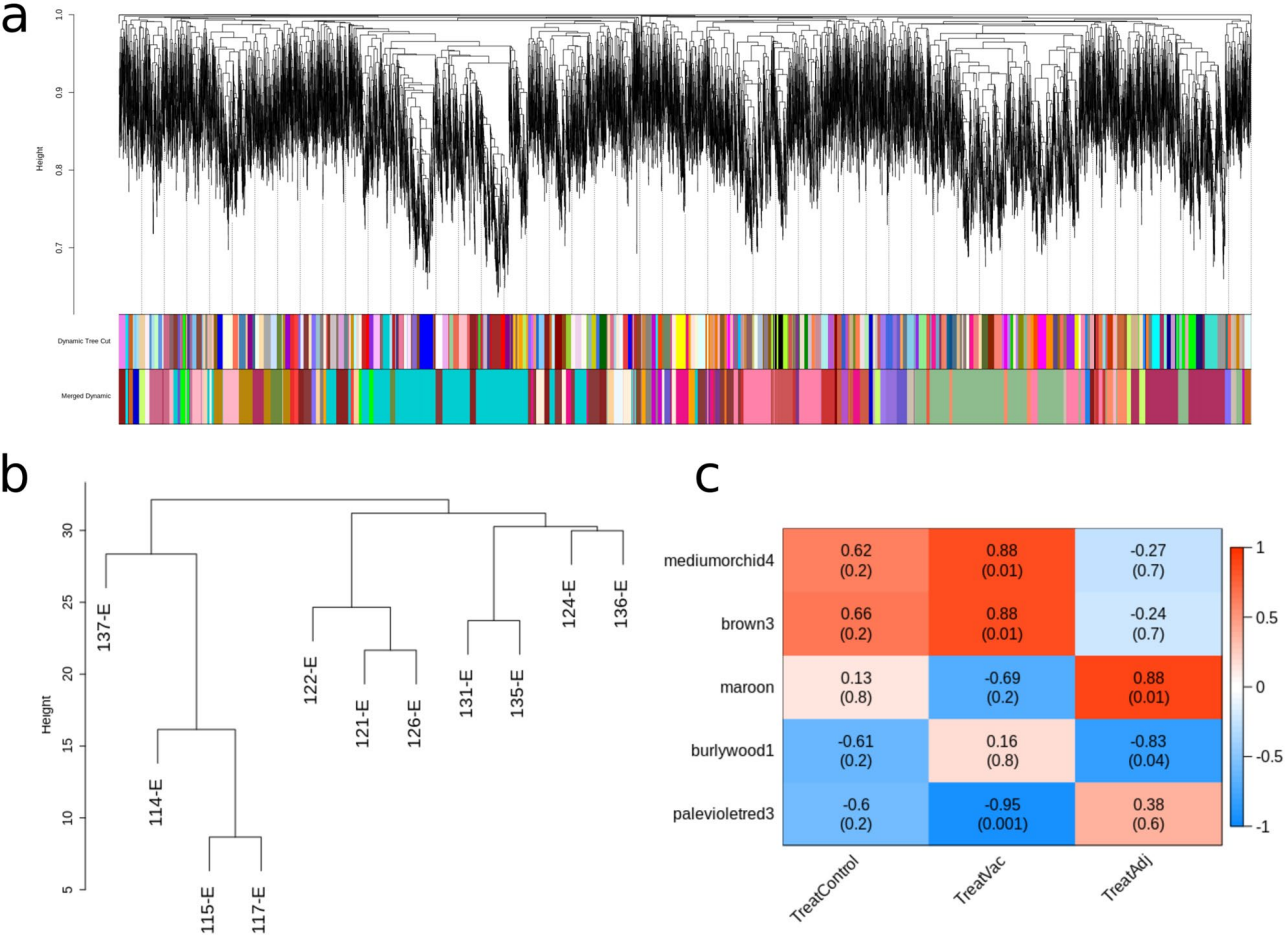
Weighted gene expression co-variance network analysis (WGCNA) summary. (**a**) Gene dendrogram obtained by average linkage hierarchical clustering. The colour rows underneath the dendrogram shows the module assignment before (Dynamic Tree Cut) and after (Merged Dynamic) modules with similar expression profiles were merged. (**b**) Hierarchical clustering of samples used in the analysis. (**c**) Module-trait associations. Each row corresponds to a module eigengene, while the columns to a trait. Each cell contains the corresponding correlations and adjusted p-values. The table is color-coded based on the correlation between the eigengene and corresponding trait. Only modules associated with at least one trait are shown.

Next, a gene co-expression network was constructed with WGCNA. Such networks provide a way to account for the coordinated expression among genes and discern possible differences between individuals that may relate to differences in treatment group. A total of 45 co-expressed gene modules were detected (Fig. 4a,b), module size ranging from 37 to 2,724 genes. Each module was assigned a ‘colour name’. We searched for significant correlations among module eigengenes and treatment parameters. There were no co-expressed modules associated with the Control group. In contrast, three modules showed strong correlations with Vac group and two with Adj group: the mediumorchid4 module (189 genes, r = 0.88, qvalue = 0.01), the brown3 module (377 genes, r = 0.88, qvalue = 0.01) and the palevioletred3 (275 genes, r = − 0.95, qvalue = 0.001) for Vac group and the maroon module (1,325 genes, r = 0.88, qvalue = 0.01) and the burlywood1 module (228 genes, r = − 0.83, qvalue = 0.04) for Adj group (Fig. 4c). Interestingly, the maroon module included 36 DEGs, the remaining modules having an insignificant number of DEGs in comparison.

The obtained treatment associated modules were further studied for enrichment of GO terms and KEGG pathways. Only the modules maroon and burlywood1 had significant enrichments, while the others, probably due to the small number of annotated genes, did not have significant enrichments. The maroon module, positively correlated with the adjuvant samples, was enriched for some GO terms, among them regulation of interleukin-1 beta production (GO:0032651), negative regulation of extrinsic apoptotic signaling pathway (GO:2001237), negative regulation of canonical Wnt signaling pathway (GO:0090090), positive regulation of immune system process (GO:0002684), and inflammasome complex (GO:0061702). A more detailed list of the enriched GO terms from the Biological Process category for the maroon module can be seen in Supplementary Fig. S3. In addition, only the maroon module was enriched in KEGG pathways, mainly: *ECM-receptor interaction* (*oas04512*), *amoebiasis* (*oas05146*), *focal adhesion* (*oas04510*), *PI3K-Akt signaling pathway* (*oas04151*), *protein digestion and absorption* (*oas04974*) and *NF-kappa B signaling pathway* (*oas04064*).

Since hub genes are likely ‘key drivers’ of the co-expression modules, we checked the treatment related modules. In Supplementary Table S1 there is a detailed list of the hub genes in these modules. To note the maroon module, in which 17 of the hub genes are DEGs. Some of them, as previously detailed, had been related with brain injury (*GSN*, *LAMA2* and *PROS1*), neuronal development (*NTN1* and *NID2*) and different diseases in brain (*MRC1* and *ANXA2*). Apart from the differentially expressed genes, there are other genes related to other functions such as insulin signalling (*INSR*, *IGFBP2* and *IGF2BP2*), blood brain barrier (*ADGRA2* and *NTN1*), ERK signalling (*INSR*, *ITGA9*, *OSMR*, *COL18A1*, *LAMA2*, *BCL2L11*, *ADAM17*, *COL4A3*, *COL4A4*, *COL4A6*, *COL2A1* and *BMP4*) and calcium signalling (*APOOL*, *HOMER3* and *TMBIM1*). It seems that the maroon module is composed of genes essential for the correct function of the brain.

**Figure 5:**
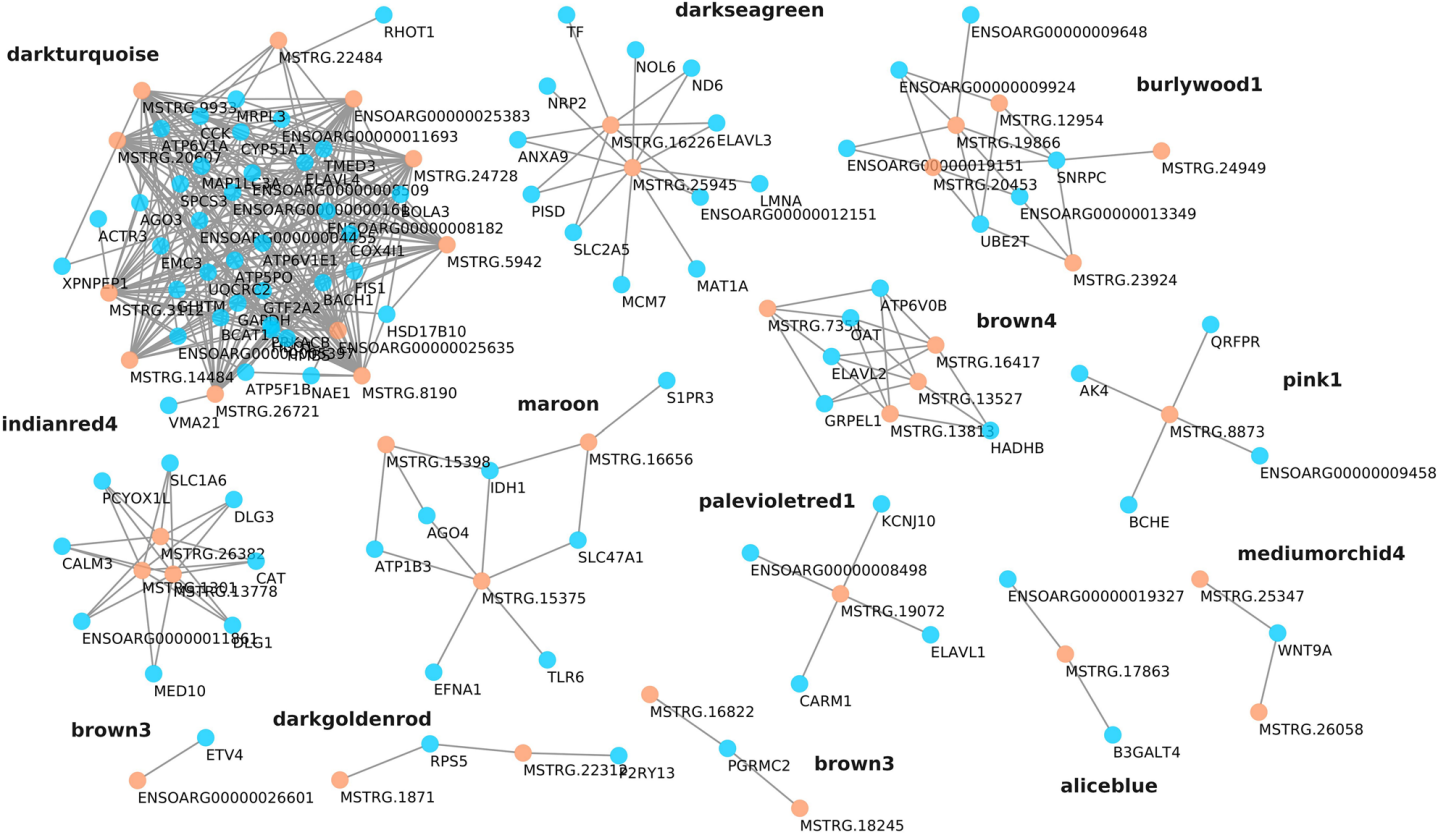
Interaction prediction of DE lncRNAs with correlated proteins (proteins with genes in the same co-expression module as the lncRNA). Interaction probability of more than 0.9 was chosen as threshold.

We performed predictions based on proposed mechanisms of action for the DE lncRNAs in the co-expression modules. Trans-acting lncRNAs could act in many ways to epigenetically regulate expression of distant genes, for instance, by recruiting or acting as scaffolds of proteins. 20,011 lncRNA-protein interactions were predicted in total, with an average of 235 interactions per lncRNA transcript. Top scoring interactions were used to build a network of lncRNA-protein interactions with proteins whose mRNA transcripts are correlated with DE lncRNAs (Fig. 5). Among these interactions appeared all four RNA-binding proteins of the ELAV/Hu family, mainly expressed in differentiated neurons.

### Statistics for miRNA-seq data

The sequenced 13 miRNA-seq libraries had an average depth of 18.2 million single-end reads. After adaptor and quality filtering, a mean of 16.3 million reads (89.55%) remained for subsequent analyses. Those reads were aligned against the *Ovis aries* reference genome (Oar3.1), allowing up to 20 multimappings per read. An average of 13.1 million reads (80.21%) were aligned to the reference.

### Analysis of differential gene expression from miRNA-seq data

After identification and prediction of miRNAs using the miRBase database, 299 miRNAs were expressed at least with one sequence read count in at least one of the 13 sample libraries. From the 299 miRNAs, 141 were annotated as *Ovis aries* miRNAs, while the others were previously annotated in other species (84 in *Capra hircus*, 20 in *Bos Taurus* and 44 in others). Ten were completely new miRNAs. A detailed list of the detected miRNAs and their sequences can be seen in Supplementary Dataset S3.

Detected miRNAs whose expression were lower than 1 CPM and found in less than four individual libraries were treated as lowly expressed miRNAs and were filtered out from the differential expression analysis. In total 259 miRNAs were used in the differential expression analysis.

**Figure 6:**
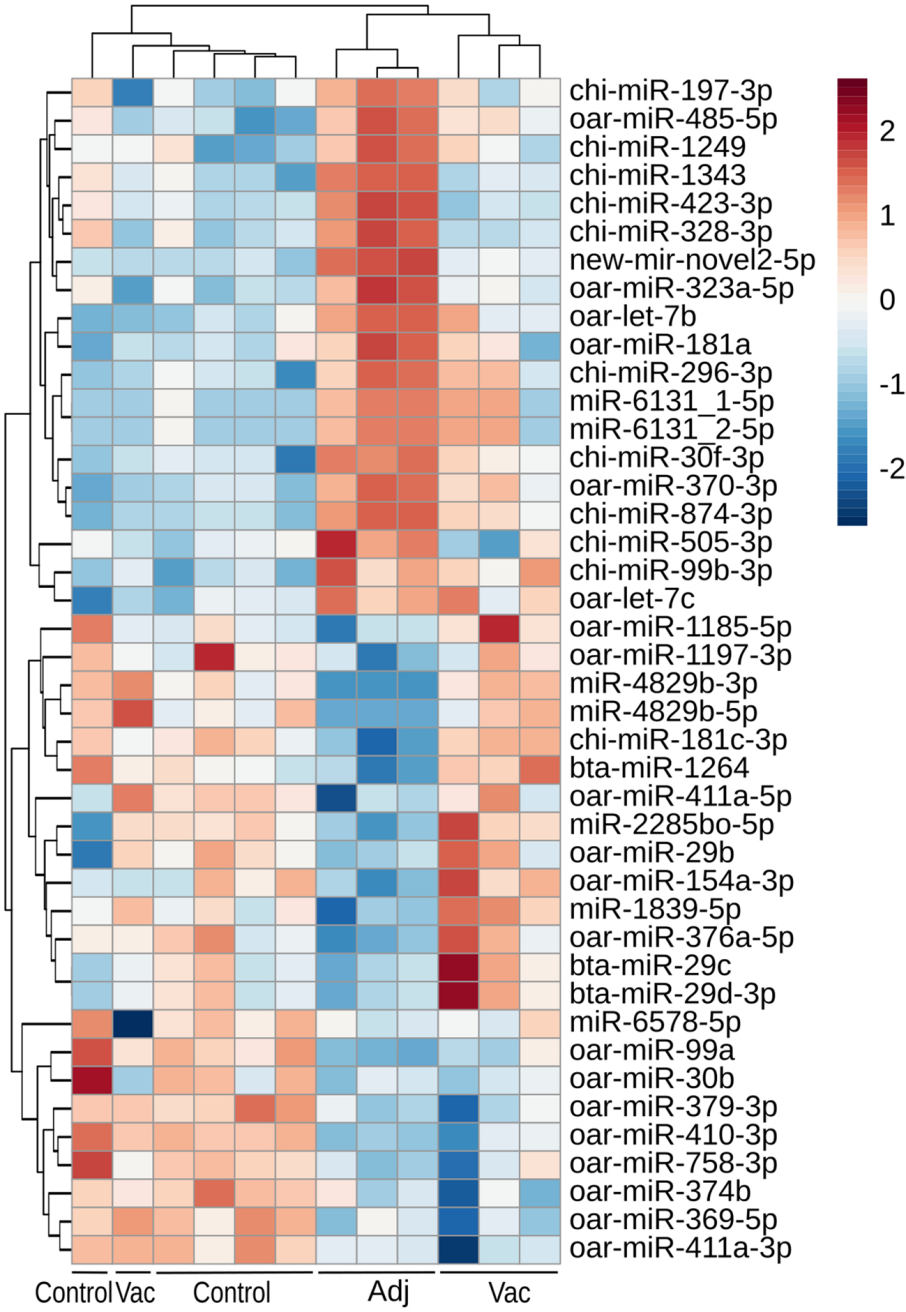
Heatmap depicting all the differentially expressed miRNAs in Adj vs. Control, Vac vs. Control and Adj vs.Vac comparisons.

A total of 38, 2 and 7 DE-miRNAs (with a p-value < 0.05 and a fold change > 1.5 or < 0.667) were identified in the Adj vs. Control, Vac vs. Control and Adj vs. Vac comparisons, respectively. All the differentially expressed miRNAs in the three comparisons are represented in a heatmap (Fig. 6) and Supplementary Table S2. Within the DE-miRNAs are factors that are clearly related to brain injury (*let-7b*, *miR-423-3p*, *miR-99b-3p*, *miR-874-3p*, *miR-29b/c*, *miR-328-3p*, *miR-99a*) and neurodegenerative diseases like AD (*miR-181c-3p*, *miR-29b/c*), PD (*miR-99b-3p*, *miR-29b/c*), ALS (*miR-181a*, *miR-30b*) and Multiple Sclerosis (MS) (*miR-369-5p*, *miR-370*, *let-7b/c*) or autoimmune diseases like lupus erythematosus (*miR-410-3p*).

### miRNA target prediction and integration of miRNA and mRNA expression profiles

Target gene predictions were performed for the differentially expressed miRNAs with three different programs (miRanda, PITA and TargetScan), taking the intersection of their results as potential targets. Two approaches were performed to integrate the miRNA and mRNA expression profiles: a correlation analysis and the isubGraph algorithm. After multiple-testing correction, nearly no pair reached significant levels in the correlated miRNA-target pairs, (Supplementary Table S3). Among the significant pairs, the majority had a positive correlation, something not expected if the miRNA acts via translational repression and/or mRNA cleavage. However, there is evidence of miRNAs enhancing translation in special cases like cell cycle arrest^12^ or mitochondrial translation^13^, but it needs to be determined whether the activation of protein translation is a general phenomenon or is only an exception. Among the negatively regulated targets, there are some genes related to the mitochondria (*ACTR10* and *MRS2*, both targeted by *let-7* family members), to maintenance of neuronal polarity and axon growth (*RUFY3*) and to apoptosis (*NAA50* and *UNC5D*).

After applying the iSubgraph algorithm, only a subgraph in the three adjuvant samples was identified (Supplementary Fig. S3), not finding any more in the remaining samples. All the pairs in the detected subgraph were positively correlated. From the subgraph stands out the miR-29 family (a family differentially expressed in Adj vs. Control and Adj vs. Vac comparisons) and their predicted targets, some of them previously related to neurodegenerative diseases (*NAV3*, a member of the neuron navigator family, and *IREB2*, which encodes a protein that is a regulator of the cellular iron metabolism). Both targets have been previously reported to be affected at protein level by the miRNA while their mRNA level remained stable in brain samples^14,15^. The miR-29c/NAV3 pair was also detected in the previous correlation analysis. The remaining elements of the subgraph are composed of miRNAs and genes not differentially expressed.

## Discussion

In this work, the molecular signature activated in the encephalon of experimentally treated sheep has been analysed for the first time. After being inoculated with either Al hydroxide containing vaccines or an equivalent amount of Al hydroxide during 16 months, the differentially expressed mRNAs, lncRNAs and miRNAs were detected and functionally characterized. Previously, the transcriptome of PBMCs had been analysed at the beginning and at the end of the experiment^8^. In this study, the same group of animals was used and their transcriptomes compared with those of control animals, which only received PBS as inoculum, at the end of the experiment. Three comparisons were made with the transcriptomes: Adjuvant inoculated vs. controls, vaccinated vs. controls and adjuvant inoculated vs. vaccinated animals.

Analysis of differential gene expression from RNA-seq data identified nearly 5 times more differentially expressed elements in the Adj vs control comparison than in the Vac vs. Control comparison. A very similar number of genes and lncRNAs differentially expressed was obtained in each comparison. The expression alteration of four genes that were previously described in other studies related to several neurological disorders were detected in this study, namely *VCAM1*, *TRPM4*, *GDF10* and *NTN1*. The first three were detected as significantly upregulated in the Adj-injected sheep, while the latter was found to be upregulated in Adj vs Control and Adj vs. Vac comparisons. VCAM1 is a cellular adhesion molecule involved in the migration of immune cells across blood–brain barrier in inflammatory central nervous system diseases^16^. *VCAM1* is also implicated in neuronal apoptosis and may play a role in the development of rheumatoid arthritis^17^ and in the pathology of intracerebral haemorrhage (ICH)^18^. *TRPM4* mediates neuronal degeneration and has been related to various neurological disorders like experimental autoimmune encephalomyelitis and MS^19^. Moreover, Li et al.^20^ found that *GDF10* was induced in peri-infarct neurons in mice, non-human primates and humans. *GDF10* is considered a stroke-induced signal that promotes axonal outgrowth and enhanced functional recovery after stroke. Finally, another gene involved in blood–brain barrier integrity, *NTN1*, was found to be upregulated in 2 comparisons. NTN1 protects the central nervous system against inflammation.

In a recent study on aluminium accumulation in different tissues of sheep in the same experiment by means of transversely heated graphite furnace atomic absorption spectroscopy, most of the accumulation values were below 1 μg/g of aluminium in encephalon. Moreover, Al content tended to be higher in the animals of the adjuvant group compared with the control group, although without reaching statistical significance^21^. The deposits of aluminium, analysed by lumogallion technique, were cell associated and sometimes closely related to vessels. In any case, the Al deposits observed in the encephalon were lower in contrast with other tissues such as lumbar spinal cord. The limited quantity of aluminium that reached this tissue could explain the low number or differentially expressed genes, comparing with other tissues such as PBMCs.

Functional characterization of the DE-mRNAs showed that there were no overrepresented GO terms in Vac vs. Control comparison. In contrast, 27 significantly overrepresented GO terms were identified in the Adj vs. Control comparison, most of them related with the mitochondrial energy metabolism*.* As Aluminium is involved in the production of reactive oxygen species (ROS), it may impair mitochondrial functions^22,23^. Changes in mitochondrial functions produce oxidative stress, leading to DNA damage and cell death. In addition, *positive regulation of cardiolipin metabolic process (GO:1900210)* and *alpha-ketoglutarate transport (GO:0015742)* GO terms were enriched in the Adj vs. Control comparison. Interestingly, cardiolipin, a phospholipid located mainly in the inner mitochondrial membrane, is associated with brain cell viability and brain homeostasis^24^. Alpha-ketoglutarate is a source of glutamate, a neurotransmitter that is involved in neurotoxicity^25^ and the transport of calcium across the inner mitochondrial membrane plays an important role in neuronal physiology and pathology^26^.

As far as lncRNAs expression is concerned, brain lncRNA expression is highly diverse, many lncRNA are brain-specific and some are associated with neural functions and diseases^27^. More than 3,000 candidate lncRNAs were identified in this work. Most of them presented characteristics previously described in sheep and other livestock species—poor sequence conservation, fewer exons than coding genes, diverse lengths and a majority of intergenic transcripts—even if they may vary depending on the classification methods^28,29^. Among the few identified conserved lncRNAs, the DE *TUNA*, downregulated in the adjuvant group, seems an interesting element. *TUNA* is required for pluripotency and neural differentiation through interactions with RNA-binding proteins in its conserved sequence^30^. It regulates *NANOG* and *SOX2* transcription factors, and *FGF4* growth factor, all of them necessary for neural differentiation.

Among the candidate lncRNAs, intronic lncRNAs showed higher correlations with their closest gene and the genes that harboured intronic lncRNAs were enriched in synaptic processes. Lately, some intronic RNAs, named stable intronic sequence RNAs (sisRNAs) have been proposed as a new layer of gene regulation. They could regulate host gene expression or act as molecular sponges for miRNAs^31^. Based on GO and KEGG analysis, our data suggest that a number of intronic lncRNAs expressed in the brain may be regulating genes that act in synapses and other signalling processes, similarly to what has been proposed for brain circRNAs^32^, which are also enriched in synaptic genes.

As previously described, a similar amount of DE coding genes and DE lncRNAs were detected. This feature is a sign of the importance of non-coding RNA classes in brain development, function and disease^33,34^. Al adjuvant treatment altered the expression of several lncRNAs, which, in turn, may alter the regulation of certain genes. Since lncRNAs have been implicated in neuronal functions in diverse ways^35^, we can predict potential mechanisms of action of lncRNAs. We used in silico predictors of lncRNA-protein interactions for the *trans* interactions. The four members of RNA-binding proteins ELAV/Hu that are mainly expressed in differentiated neurons are in the top predictions. *ELAVL4*, for instance, interacts with many mRNAs altering translation efficiency and stability, and is related to neuronal differentiation, self-renewal and plasticity^36^. Their activity could be altered by competing RNAs (ceRNAs) like other mRNAs or lncRNAs^37^. In fact, recent studies show that *ELAVL1* interacts with several lncRNAs in mice and could have a role in neural stem cell differentiation^38^.

A co-expression analysis was also performed for mRNAs and lncRNAs with WGCNA software, and 45 different modules were obtained. Interestingly, 5 of them correlated with different treatments, that is, 3 modules correlated with Vaccinated group (mediumorchid4, brown3 and palevioletred3) and 2 with Adjuvant group (maroon and burlywood1). Among them, the maroon module contained 36 DEGs and showed significant enrichments in specific KEGG pathways. Xu et al.^7^ also found that *ECM-receptor interaction, protein digestion and absorption, focal adhesion and PI3K-Akt signaling pathway* were significantly enriched in the hippocampus of Al-treated rats. Among these pathways, the *PI3K-Akt signaling pathway* is expressed during central nervous system development^39^ and it is well known that this pathway is particularly important for mediating neuronal survival, differentiation and metabolism^40^. In addition, focal adhesion and ECM-receptor interaction signalling are known to be involved in the regulation of synaptic plasticity^7,41^ and NF-κB pathway plays a crucial role on neurogenesis, cellular responses to neurological injury and neuroinflammation^42,43^. Currently, there are few reports regarding the role that these pathways play in the neurotoxicity caused by aluminium.

As far as the expression analysis of microRNAs is concerned, a dysregulation in the miRNAome of the adjuvant group is shown, whilst nearly any significant change was detected in the vaccinated group. Among the 42 differentially expressed miRNAs, there were some previously described in other studies related to neurological diseases. *let-7b* was found upregulated in the adjuvanted group of animals in this work. This miRNA is an activator of the TLR7 signalling pathway, which leads to neurodegeneration^44^. In addition, *miR-374b* and *miR-30b* expression was decreased, in the adjuvanted group. Those miRNAs show an expression pattern similar to the one detected in patients with sporadic ALS^45,46^.

Apart from neurodegenerative diseases, some differentially expressed miRNAs in this work had been previously described in studies related to brain injury. The expression levels of *miR-874-3p* and *miR-423-3p* were increased and the expression levels of *miR-99a* and *miR-29c* were decreased in the adjuvant group. *miR-874-3p* expression has been reported to increase after injury in neurons and his over-expression leads to increased stress and vulnerability, affecting inflammatory and apoptotic processes^47^. In contrast, *miR-423-3p* might be compensatorily over-expressed in response to apoptosis and exert anti-apoptotic effects in chronic temporal lobe epilepsy^48^. Both *miR-99a* and *miR-29c* have been involved in oxidative stress and apoptosis^49,50^. Overall, the miRNA expression analysis has linked apoptotic pathways, mitochondrial dysfunction and ECM related pathways to the intensive vaccination with the adjuvant alone.

Lastly, mRNA and miRNA expression profiles were integrated. Within the negatively regulated targets of the differentially expressed miRNAs there are genes related to mitochondrial function, maintenance of neural polarity and DNA damage control. Mitochondrial transport is crucial for the function of the nervous system due to the particular cellular morphology of neurons and the need to supply energy to remote regions^51^. *ACTR10*, which is a negatively regulated and predicted target of *let-7b*, is part of the dynactin complex and absence of the protein encoded by this gene has been shown to disrupt mitochondrial retrograde transport, leading to accumulation of mitochondria in axon terminals^52^. In addition, mitochondria are one of the major pools of intracellular Mg and its deficiency seems to be related to mitochondrial dysfunction. *MRS2*, which is other predicted target of *let-7b*, is a mitochondrial Mg transporter that has been related to defects in the organelle and apoptosis^53^. It should be pointed out that generally miRNAs function in the cell cytoplasm, but there is evidence of miRNAs located in other locations, being the *let-7* family one of the miRNAs found in mitochondria cytoplasm^54^. In adjuvant-only vaccinated animals Al might be causing an imbalance in metal ion levels, among them Mg^2+^, something that has been seen in rats treated with an intragastric administration of Al gluconate^55^.

Al hydroxide alone altered the expression of different mRNAs as well as lncRNAs and miRNAs important for neuronal cell survival, mitochondrial energy metabolism, metal ion balance and others associated with neurological disorders. This work is based on a long term experiment using sheep as a model. Although a considerable amount of aluminium was inoculated in a relative short period of time, the fact that certain Al salts are able to impair gene expression in a way that suggests neurotoxicity in this model should be taken into account for the production of safer vaccines.

## Materials and methods

### Animals

The animals studied in this work were previously analysed for a different tissue (PBMCs)^8^. Briefly, twenty-one Rasa Aragonesa purebred lambs were selected from a single pedigree flock of certified good health at three months of age and did not undergo any vaccination before the experiment. The flock analysed in this study was stablished at the experimental farm of the University of Zaragoza, with ideal controlled conditions of housing, management and diet. The experiment started after an acclimatization phase of two months, when the animals were five months old. For the purpose of the present work, they were randomly distributed in different treatment groups, n = 7 each. Each treatment group was kept isolated from the others in three adjacent identical home pens with the same conditions of housing, diet and management across all the study. Each group received a parallel subcutaneous treatment with either commercial vaccines containing Al hydroxide [Al (OH3)_3_] as adjuvant (Group Vac), Al hydroxide only (Group Adj; Alhydrogel^®^, CZ Veterinaria, Spain) or PBS (Group Control). Nine different vaccines were used and a total of 19 inoculations were applied to each animal throughout 16 different inoculation dates, thus entailing a total amount of 81.29 mg of Al per animal in Vac and Adj groups. The complete study lasted 475 days, from February 2015 to June 2016. Supplementary Table S4 includes details of the commercial vaccines used and the inoculation protocol applied. Twelve animals were included for the RNA-seq analysis, 4 of each treatment group. For the validation of the sequencing data 9 different animals were included, 3 of each treatment group (Table 1).

**Table 1:** Samples used in RNA-seq and RT-qPCR study.

Treatment	Animals (12)	Samples
**RNA-seq**		
Aluminum	4	114-E, 115-E, 116-E, 117-E
Vaccine	4	121-E, 122-E, 124-E, 126-E
Control	4	131-E, 135-E, 136-E, 137-E

Treatment	Animals (9)	Samples
**RT-qPCR**		
Aluminum	3	111-E, 112-E, 113-E
Vaccine	3	123-E, 125-E, 127-E
Control	3	132-E, 133-E, 134-E

For miRNA-seq 133-E sample was also sequenced.

### Tissue collection and RNA extraction

Tissues for pathologic studies were collected at necropsy. Samples of 1 g of parietal lobe from each sheep, with constant proportions of gray and white matter, were taken for RNA extraction and preserved in RNAlater solution (Ambion, Austin, TX, USA) at − 80 °C. The experimental procedure to obtain RNA was similar to the one previously performed in the analysis of PBMCs^8^. Total RNA was isolated from encephalon tissue using TRIzol Reagent (Invitrogen, Carlsbad, CA, USA) and PureLink RNA Mini Kit (Invitrogen). 60 mg tissue samples were homogenized in 1 ml of TRIzol Reagent using Precellys^®^24 homogenizer (Bertin Technologies, Montigny-le-Bretonneux, France) combined with 1.4 and 2.8 mm ceramic beads mix lysing tubes (Bertin Technologies). RNA isolation was performed following manufacturer instructions and RNA was suspended in RNase free water and stored at − 80 °C. RNA quantity and purity was assessed with NanoDrop 1000 Spectrophotometer (Thermo Scientific Inc, Bremen, Germany). RNA integrity was assessed on a Agilent 2100 Bioanalyzer with Agilent RNA 6000 Nano chips (Agilent Technologies, Santa Clara, CA, USA), which estimates the 28S/18S (ribosomic RNAs) ratio and the RNA integrity number (RIN value). The samples presented an average RIN value of 8.06 and a 260/280 ratio > 1.7.

### RNA sequencing

The TruSeq Stranded Total RNA kit with Ribo-Zero (Illumina, San Diego, CA, USA) and the TruSeq Small RNA library prep kit (Illumina) were used for Total RNA-seq and miRNA-seq, respectively. Total RNA libraries were sequenced on a HiSeq2000 with a mean sequencing depth of 75 million reads (75 bp paired-end reads) at CNAG (Centro Nacional de Análisis Genómico, Barcelona, Spain), while miRNA libraries were sequenced on a HiSeq2500 with a mean sequencing depth of 19 million reads (50 bp single-end reads) at CRG (Centro de Regulación Genómica, Barcelona, Spain). The samples used for sequencing and qPCR can be seen in Table 1.

### Total RNA expression analysis

The bioinformatics procedure to obtain the expression matrix was similar to the one previously described in the analysis of PBMCs^8^. Briefly, after quality filtering and trimming, the reads were aligned with the STAR algorithm [v2.5.4a]^56^ to the *Ovis aries* genome build Oar3.1^57^. For each library, the uniquely aligned fragments were assigned to annotated genes in a strand specific manner with featureCounts [v1.6.0]^58^. Apart from annotated genes, one of the interests of this work is to find new lncRNAs and study their function in sheep brain. For that purpose, an additional step after mapping was necessary. The StringTie [v1.3.3b]^59^ transcriptome assembler was used to reconstruct the transcriptome from the previous mapping. From this assembly, only candidate lncRNAs were selected (the selection process and analysis is explained below) and their counts were added to the count matrix of annotated genes.

The same sample (116-E) was treated as outlier and was filtered out from the analysis. Prior to the differential expression, the SVA package [v3.26.0]^60^ was applied to remove unwanted variation and the obtained surrogate variables were incorporated into the testing model. A PCA was obtained with the corrected data (Supplementary Fig. S1A). In this PCA the samples grouped according to treatment condition. The differential expression analysis was performed using DESeq2 [v1.18.1]^61^ with the following variables in the model: *treatment* (Control, complete vaccine [Vac] or adjuvant only [Adj]) and *SVA covariates (*surrogate variables calculated by sva). Three different comparisons were made (Adj vs. Control, Vac vs. Control and Adj vs. Vac) in which differentially expressed genes (DEGs) were selected as those with an adjusted p-value (with the Benjamini Hochberg method) threshold of < 0.05 and a fold change > 1.5 or < 0.667. Then, gene enrichment analyses were conducted using the GO database in PANTHER [v12.0]^62^ and the KEGG database in DAVID [v6.8]^63^, considering enriched terms as those with an adjusted p-value threshold of < 0.05.

### Weighted gene co-expression network analysis

A weighted gene co-expression network analysis was performed using the WGCNA [v1.63]^64,65^ R package. Briefly, the similarity matrix was constructed from the normalized data using absolute values of the biweight midcorrelation, chosen for being more robust against outliers. Then, the adjacency matrix was defined by raising the similarity matrix to a power β. The parameter β was selected based on the minimum value required to get a scale-free topology network (R^2^ > 0.8), in our data being β = 28. Once the network was constructed, module (clusters of densely interconnected genes) detection was the next step, setting a minimum module size of 30 genes. Finally, modules with similar expression profiles were merged based on a height cut-off threshold of 0.3.

Next, we sought modules with strong correlations with the treatment groups. For that purpose, the treatment variable was dichotomized in all possible combinations (one group against the other two). For each of the identified modules, eigengene values (the first principal component of each module) were generated and were used as representation of the weighted average of the gene expression profile in the modules. Pearson correlations and their associated p-values were generated for all pairwise comparisons of the module eigengene expression values and the treatment parameters. All the p-values were used for estimation of the FDR (q-value) with the *qvalue* R package, selecting those modules with a q-value threshold < 0.05.

Modules exhibiting high correlation with the treatment were further studied for enrichment of GO terms and KEGG pathways, considering statistically significant those with an adjusted p-value threshold of < 0.05. Apart from enrichment analysis, the hub genes of each module were obtained. For that purpose, the module membership (MM) and gene significance (GS) values were calculated. GS values are the Pearson correlations between the single expression value of each gene and the treatment parameter, whilst MM values are the Pearson correlations between the single expression value of each gene and module eigengene values. We defined hub genes as those belonging to the ≥ 85th percentile for both MM and GS in each module^66^. Those genes are likely ‘key drivers’ and might play important roles in the treatment.

### Analysis of lncRNAs

gffcompare software was used to classify all sequenced transcripts based on their location relative to the annotation and extract unknown intergenic transcripts (lincRNAs), intronic lncRNAs and antisense lncRNAs. Multiexonic transcripts of less than 200 nucleotides and single-exon transcripts of less than 2,000 nucleotides were filtered out. The coding potential of the remaining transcripts was assessed with three approaches. Coding Potential Calculator 2 (CPC2) is a machine learning based program with a species-neutral model able to classify coding and non-coding sequences^67^. Coding-Potential Assessment Tool (CPAT) is another machine learning based program that we trained and selected the classification threshold following authors’ instructions using available bovine coding and non-coding sequences^68^. HMMER 3.1b2^69^ was used to detect Pfam^70^ protein domains in our potential lncRNAs, which were translated into the three possible frames. Transcripts classified as non-coding by CPC2 and CPAT and without protein domains detected were selected and treated as lncRNAs for their functional analysis. Besides, genes already annotated in sheep (Oar_v3.1) with “lincRNA” biotype were also added. To evaluate the sequence conservation and to look for known homologues we performed a Blast search with each lncRNA transcript to the entire RNAcentral database, which has an up-to-date collection of non-coding RNA sequences^71^.

For *trans* acting lncRNAs potential protein-interacting lncRNAs were predicted with LncADeep tool^72^ and sequences of proteins with at least evidence at transcript level or from homology were downloaded from UniProt. For more confident results, interactions were only predicted for proteins from genes in the same co-expression modules and a probability of 0.9 was set as threshold.

### MicroRNA expression analysis

The procedure to analyse the miRNAs is similar to the one previously described in the analysis of PBMCs in the same group of animals^8^. Briefly, after adaptor removal and quality filtering, some of the sRNAtoolboxVM^73^ modules were applied. First, the sRNAbench module was used to align sequences to the *Ovis aries* reference genome Oar3.1, to profile the expression of small RNAs and to predict novel miRNAs. (searching for human, mouse, cow and goat homologous miRNA sequences). Then, the differential expression analysis was performed using DESeq2 with the same model as for Total RNA-seq, applying first the SVA package to remove unwanted variation. Similar to the RNA-seq analysis, the same sample (116-E) was treated as outlier and was filtered out from the analysis. A PCA was obtained with the corrected data (Supplementary Fig. S1B) where samples were grouped according to treatment condition. The differentially expressed miRNAs were selected as those with an adjusted p-value (with the Benjamini–Hochberg method) threshold of < 0.05 and a fold change > 1.5 or < 0.667.

The mRNAconsTarget module was used to identify potential miRNA target genes with miRanda^74^ and PITA^75^ algorithms. At the same time, the target prediction algorithm TargetScan^76^ was applied independently. To reduce false positives and select candidate targets, only those genes that were common across the three programs were selected for further analysis.

### Integration of miRNA and mRNA expression profiles

The miRNA and mRNA data were integrated following the same procedure as in our previous work of PBMCs^8^. Correlations between miRNA and mRNA expression values were determined using the R statistical software [v3.5.0]. A test for association between paired samples using the Spearman’s rank correlation coefficient was applied with the R *cor.test* function. The obtained p-values were used for estimation of the FDR (q-value) with the *qvalue* R package, using a threshold of < 0.05 to indicate significant miRNA-mRNA pairs. Apart from the correlation analysis, in an attempt to discover miRNA-gene patterns, a subgraph mining tool was applied. For that purpose, the iSubgraph^77^ algorithm was used, which searches for frequent cooperative regulations of genes and miRNAs happening in a minimum group of samples. The parameters were set as follow: the threshold for Up and Down tags was set at 0.75; and to report a pattern, that pattern needed to be found at least in three samples.

### Validation of differential mRNAs by qPCR

To validate changes identified by RNA-seq experiments, the relative expression levels of 13 mRNAs that were selected based on significant changes seen in the RNA-seq analyses were verified by qPCR. The strategy followed was similar to the one previously done for PBMCs samples^8^. Briefly, primers were designed using the PrimerQuest and OligoAnalyzer tools of Integrated DNA Technologies (IDT). All primers used for real time PCR experiments are listed in Supplementary Table S5. Quantitative PCR amplifications were performed using PowerUp™ SYBR™ Green Master Mix (Applied Biosystem, Foster City, CA, USA) in a 10 µl final volume reaction on a QuantStudio^®^ 3 detection system (Applied Biosystem). The conditions were as follows: 1 cycle of 50 ℃ for 2 min, 1 cycle of 95 ℃ for 2 min, 40 cycles of denaturation at 95 ℃ for 15 s, annealing at 60 ℃ for 60 s, and a dissociation curve to measure the specificity of the amplification. The stability of candidate endogenous control was analysed using GenEx software of MultiD [v5.4] (NormFinder^78^ and GeNorm^79^ algorithms). *HPRT* and *ATP1A1* were the two most stable genes, so these two reference genes were used as an internal control to normalize the data. The expression level of mRNA transcripts was calculated using the 2^−Δ(ΔCt)^ method. Statistical significance of the comparison between results obtained with RNA-seq and RT-qPCR was calculated by using t-test. In all analyses, differences were considered significant when p values were < 0.05.

### Ethics statement

All experimental procedures were approved and licensed by the Ethical Committee of the University of Zaragoza (ref: PI15/14). Methods were carried out under the following guidelines: Spanish Policy for Animal Protection (RED53/2013) and the European Union Directive 2010/63 on protection of experimental animals.

## Supplementary information

Supplementary Dataset 1.

Supplementary Dataset 2.

Supplementary Dataset 3.

Supplementary Information.

## Data Availability

The data discussed in the publication have been deposited in NCBI’s Gene Expression Omnibus (GEO) and are accessible through GEO Series accession number GSE128597 (https://www.ncbi.nlm.nih.gov/geo/query/acc.cgi?acc=GSE128597).
