# Supplementary material for: Whole transcriptome approach to evaluate the effect of aluminium hydroxide in ovine encephalon
Source: Sci Rep. 2020 Sep 17;10:15240. doi: 10.1038/s41598-020-71905-y (PMC7498608; doi:10.1038/s41598-020-71905-y)
Supplement: Supplementary file 4 — Supplementary Information. [file 41598_2020_71905_MOESM4_ESM.pdf]

Supplementary Material

Whole transcriptome approach to evaluate the effect of Aluminium hydroxide in ovine encephalon

Endika Varela-Martínez[1,#], Martin Bilbao-Arribas[1,#], Naiara Abendaño[1], Javier Asín[2], Marta Pérez[2], Damián de Andrés[3], Lluís Luján[2] and Begoña M. Jugo[1*]

[1]Department of Genetics, Physical Anthropology and Animal Physiology, Faculty of Science and Technology, University of the Basque Country (UPV/EHU), Leioa, Spain

[2]Department of Animal Pathology, University of Zaragoza, Zaragoza, Spain

[3]Institute of Agrobiotechnology (CSIC-UPNA-Gov. Navarra), Navarra, Spain

[#] These authors contributed equally to this work.

*** Correspondence:**
Begoña M. Jugo
e-mail: begonamarina.jugo@ehu.eus

1 Supplementary Figures (S1, S2 and S3)

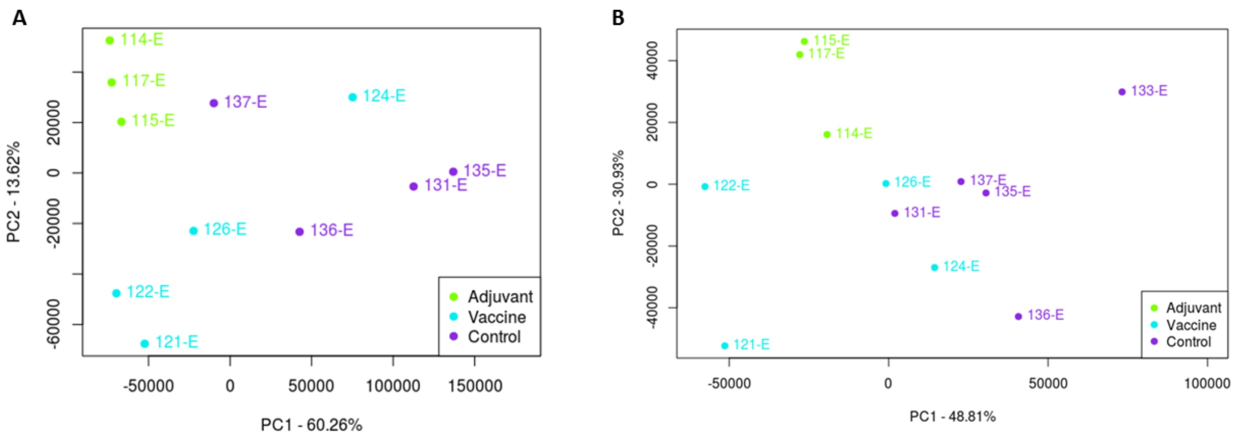

Figure S1: PCA (Principal Component Analysis) plots of Total RNA-seq and miRNA-seq data from animals treated with commercial vaccines (Vaccine group), with the adjuvant only (Adjuvant group) or with PBS (Control group). (A) Total RNA-seq PCA plot after outlier removal and batch effect correction with SVA generated from 16,369 genes that passed the filtering criteria. (B) miRNA-seq PCA plot after outlier removal and batch effect correction with SVA generated from 259 miRNAs that passed the filtering criteria.

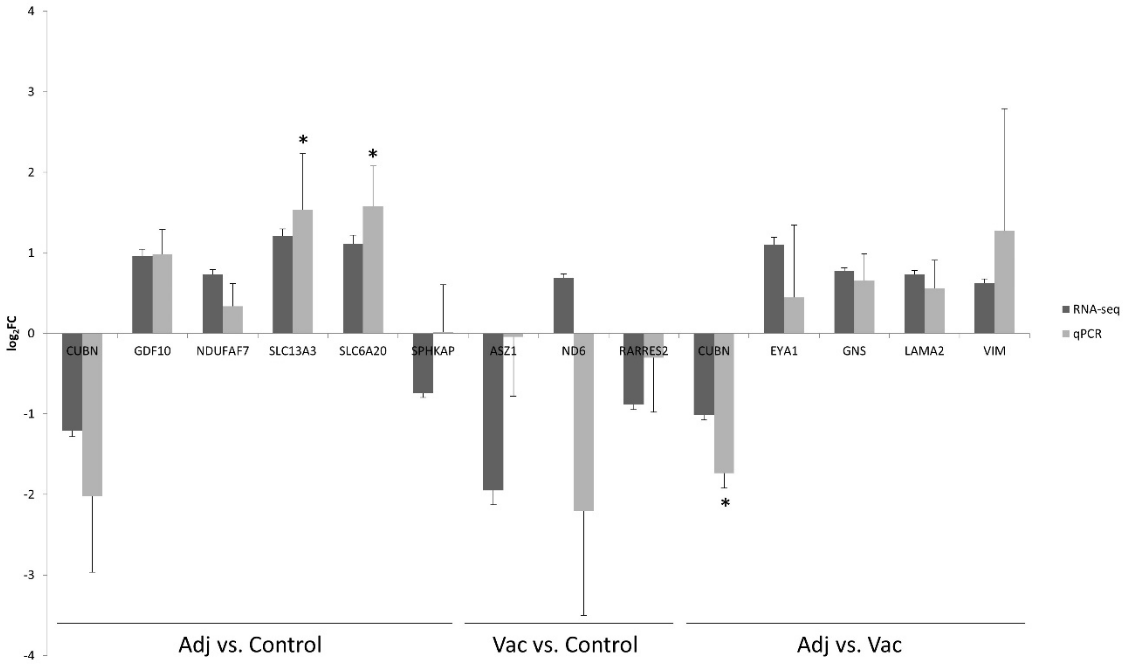

Figure S2: Validation of the RNA-seq data for the mRNAs by RT-qPCR. Log_2FC in Adj vs. Control, Vac vs. Control and Adj vs. Vac comparisons are shown. Error bars correspond to the standard error of the mean (SEM). Statistically significant differences in the expression of mRNAs measured by RT-qPCR are shown with an asterisk ($p < 0.05$).

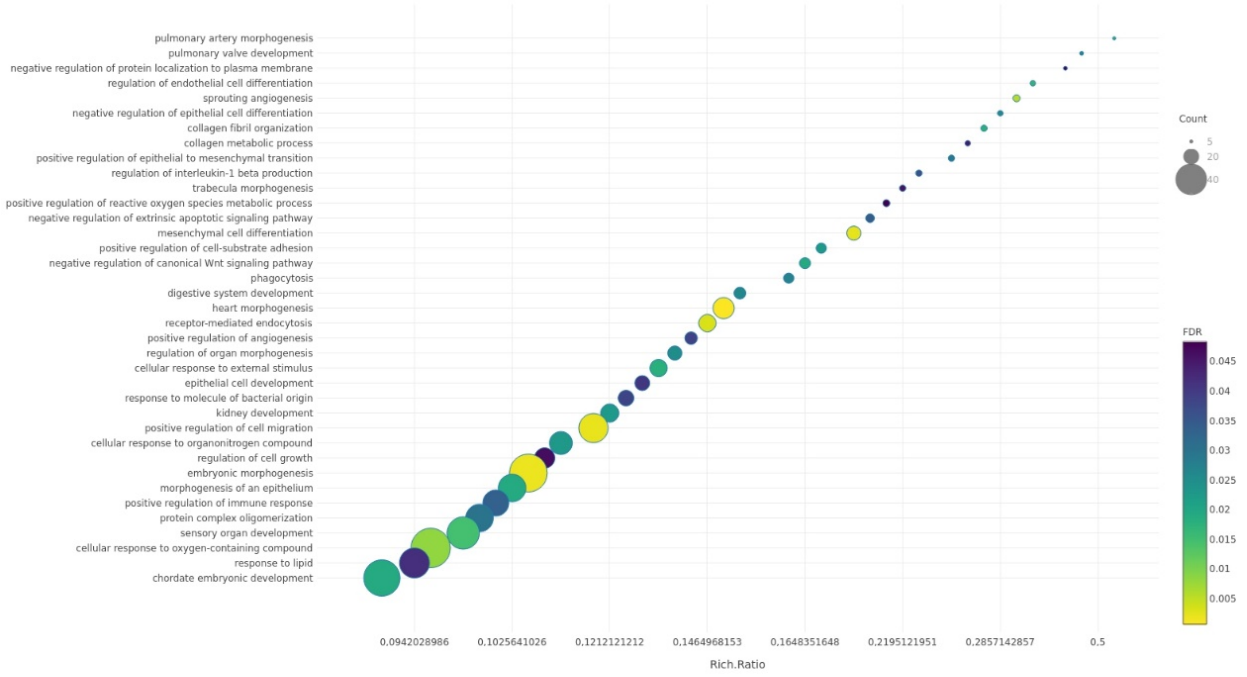

Figure S3: GO enrichment term analysis from the Biological Process category in the maroon module. The bubble plot shows in the Y-axis the enriched GO terms, while in the X-axis the rich ratio is represented (rich ratio=amount of differentially expressed genes in the term/all genes included in the term). Size and color of the bubble represents the number of differentially expressed genes in the GO term and enrichment significance (FDR), respectively.

Table S1: List of hub genes (defined as those belonging to the ≥85th percentile for both Module Membership and Gene Significance) in each module related to a treatment group.

Module	Hub Genes
mediumorchid4	SCML4, PROKR2, TTC25, HHLA2, MSTRG.16709, MSTRG.25347, MSTRG.25465, MSTRG.8370
brown3	KRBA1, MAST2, RET, RUNDC1, FLRT1, ABLIM3, SMG5, PODN, TBR1, MROH2B, GABBR2, SIRT4, THRA, TENM2, NKAP, MCTP1, SOWAHB, CCKBR, ENSOARG00000001628, ENSOARG00000013353, ENSOARG00000017276, MSTRG.12153, MSTRG.3307, MSTRG.3391
palevioletred3	RARRES2, ATPAF2, AKR1A1, PDGFD, NQO1, RNF187, TYROBP, MTFR1L, IL6ST, NET1, TMEM186, ACAT1, GNE, NECAP2, SCARF1, GNG5, TCIRG1, CH25H, C2orf69, TTC32, HERPUD1, PLA1A, GNL2, ENSOARG00000010890, MSTRG.17597, MSTRG.22964, MSTRG.25877
maroon	INSR, SLC22A6, ITGA9, ADGRA2, TFPI2, CAV1, SNX33, APOOL, MYOF, HPSE, NTN1, RASSF3, CSGALNACT1, ACSF2, KCNT1, TRIP10, STOX1, MOCOS, ZIC4, GSN, PLEKHH2, GHR, TIMELESS, COLEC12, DAB2, TNFRSF11B, OSMR, TTC23, HOMER3, MYOM1, SUCLG2, SPTLC3, SLC6A13, ISYNA1, MRC1, SLC4A5, COL18A1, C1orf115, NEXN, LAMA2, CROT, BCL2L11, SVIL, FIBIN, ADAM17, DSN1, PROS1, XPNPEP3, CILP, COL4A6, ALDH7A1, SNX24, COL2A1, KCNJ13, IGFBP2, TMBIM1, FSTL1, FAM43A, COL4A4, COL4A3, IGF2BP2, EHHADH, NID2, ANXA2, BMP4, L3HYPDH, ENSOARG00000002862, ENSOARG00000002951, ENSOARG00000003361, ENSOARG00000003373, ENSOARG00000008491, ENSOARG00000009919, ENSOARG00000016627, ENSOARG00000018242, ENSOARG00000018303, MSTRG.10448, MSTRG.20274, MSTRG.22052, MSTRG.8167, MSTRG.8811
burlywood1	RGS17, ST6GALNAC5, DPCD, ENSOARG00000001664, ENSOARG00000002028, ENSOARG00000002172, ENSOARG00000005831, ENSOARG00000013502, ENSOARG00000014615, ENSOARG00000019256, MSTRG.19866

Table S2: Differential expression results from the miRNA-seq in three different comparisons. Only miRNAs with FDR-adjusted P values of ≤ 0.05 in DESeq2 are shown.

miRNA	log2FoldChange	padj
**Vaccine VS Control**		
oar-miR-374b	-1,603	1,51E-04
chi-miR-30f-3p	0,969	4,75E-02
**Adjuvant VS Control**		
chi-miR-181c-3p	-1,128	1,09E-07
oar-miR-374b	-1,824	1,09E-07
miR-1839-5p	-3,371	1,85E-06
oar-miR-411a-5p	-1,295	1,80E-05
chi-miR-30f-3p	1,234	1,30E-04
oar-miR-369-5p	-1,414	7,48E-04
oar-miR-410-3p	-1,012	7,48E-04
miR-2285bo-5p	-3,685	7,48E-04
oar-let-7b	0,834	1,52E-03
chi-miR-423-3p	0,744	2,95E-03
new-mir-novel2-5p	0,961	3,91E-03
oar-miR-370-3p	1,131	4,29E-03
chi-miR-99b-3p	0,795	4,79E-03
oar-miR-1197-3p	-0,95	5,53E-03
bta-miR-1264	-1,421	6,02E-03
chi-miR-874-3p	0,73	8,80E-03
bta-miR-29c	-1,122	1,12E-02
bta-miR-29d-3p	-1,122	1,12E-02
oar-miR-485-5p	0,784	1,12E-02
chi-miR-296-3p	1,181	1,17E-02
oar-miR-29b	-1,051	1,17E-02
oar-miR-181a	0,934	1,31E-02
chi-miR-328-3p	0,711	1,53E-02
oar-miR-99a	-0,679	1,80E-02
oar-miR-30b	-0,875	2,33E-02
chi-miR-1249	1,005	2,52E-02
oar-miR-154a-3p	-1,006	2,56E-02
oar-let-7c	0,606	2,84E-02
oar-miR-376a-5p	-1,367	3,00E-02
oar-miR-379-3p	-1,271	3,00E-02
oar-miR-1185-5p	-2,831	3,56E-02
chi-miR-1343	0,653	3,67E-02
chi-miR-197-3p	0,646	3,67E-02
oar-miR-758-3p	-0,801	3,67E-02
oar-miR-323a-5p	0,805	3,92E-02
oar-miR-411a-3p	-0,915	4,08E-02
miR-6578-5p	-4,17	4,66E-02
chi-miR-505-3p	0,775	4,79E-02

Table S2: (continued).

miRNA	log2FoldChange	padj
**Adjuvant VS Vaccine**		
chi-miR-181c-3p	-1,138	6,80E-05
miR-1839-5p	-2,855	3,88E-03
bta-miR-29c	-1,409	2,12E-02
bta-miR-29d-3p	-1,409	2,12E-02
oar-miR-411a-5p	-0,975	4,95E-02
chi-miR-423-3p	0,747	4,95E-02
miR-2285bo-5p	-3,152	4,95E-02

Table S3: Significant miRNA-targets correlations after multiple testing correction. In bold the negatively correlated pairs.

miRNA	Transcript	rho	padj
let-7b	ACTR10	**-0,84**	0,0484
let-7b	CEP135	0,93	0,0163
let-7b	GGH	**-0,86**	0,0377
let-7b	PALD1	0,85	0,0415
let-7b	RUFY3	**-0,90**	0,0247
let-7c	ACTR10	**-0,85**	0,0415
let-7c	FBXL12	0,85	0,0445
let-7c	MRS2	**-0,84**	0,0484
let-7c	SLC20A1	**-0,91**	0,0217
miR-181c-3p	ZDHHC2	0,89	0,0262
miR-197-3p	GDF11	0,90	0,0247
miR-197-3p	NAA50	**-0,89**	0,0262
miR-197-3p	PM20D2	**-0,89**	0,0262
miR-197-3p	RIN2	0,86	0,0377
miR-29c	AP2B1	0,86	0,0377
miR-29c	ASXL3	0,95	0,0031
miR-29c	MARCH9	0,85	0,0445
miR-29c	NAV3	0,85	0,0415
miR-29d-3p	AP2B1	0,86	0,0377
miR-29d-3p	ASXL3	0,95	0,0031
miR-29d-3p	MARCH9	0,85	0,0445
miR-29d-3p	NAV3	0,85	0,0415
miR-30b	JOSD1	0,85	0,0445
miR-30f-3p	FAM167A	0,84	0,0484
miR-30f-3p	MRPS18C	**-0,86**	0,0377
miR-30f-3p	SIGLEC1	0,84	0,0484
miR-30f-3p	SIRT2	0,88	0,0313
miR-323a-5p	NAT10	0,87	0,0377
miR-379-3p	LAP3	**-0,84**	0,0484
miR-379-3p	NDST1	**-0,86**	0,0377
miR-410-3p	UNC5D	**-0,88**	0,0313
miR-485-5p	MRPS27	**-0,85**	0,0445
miR-485-5p	SEC61A1	0,91	0,0217
miR-485-5p	SLC36A1	0,92	0,0205

Table S4: Description of the vaccines used. Aluminium content was established by inductively coupled plasma atomic emission spectrometry and calculated per total dose.

Vaccine number	Commercial name	Manufacturer	Antigen/s	Inoculation day	mg of Al per dose
1	Heptavac P Plus	MSD Animal Health S.L.	Pasteurella multocida, Mannheimia haemolytica, Clostridium spp.	0, 23, 233	7.5
2	Autogenous vaccine	Exopol	Staphylococcus aureus spp. Anaerobius	44, 69, 349	1.64
3	Vanguard R	Zoetis	Rabies virus	98	1.03
4	Agalaxipra	Hipra	Mycoplasma agalactiae	129, 146	6.76
5	Ovivac CS	Hipra	Chlamydophila abortus, Salmonella abortus ovis	209, 233	5.60
6	Autogenous vaccine	Exopol	Corynebacterium pseudotuberculosis	254, 272	1.32
7	Bluevac-1	CZ Veterinaria S.A.	Bluetongue virus serotype 1	293, 329	4.18
8	Bluevac-4	CZ Veterinaria S.A.	Bluetongue virus serotype 4	293, 329	4.16
9	Bluevac BTV 8	CZ Veterinaria S.A.	Bluetongue virus serotype 8	449, 470	4.40

Table S5: List of the selected mRNAs and the corresponding primer sequences for the validation of the RNA-seq.

Gene	GenBank	Primer code	Bases	Sequence (5´-3´)	Amplicon size
**Target Genes**					
CUBN	XM_015099599.1	CUBN-F	23	ATCCAAATATGATGACTGTGAGG	99
		CUBN-R	17	CTGTACTCGGGCTCTCC	
GDF10	XM_004021551.3	GDF10-F	21	GGACATAGGGTGGAATGAGTG	95
		GDF10-R	18	GGACCATCTTGGGCATCG	
NDUFAF7	XM_004006018.3	NDUFAF7-F	18	GCAGCTTTCCAACTGGTG	80
		NDUFAF7-R	24	CCAAGTTGACTAAATACCCTCAAA	
SLC13A3	XM_004014618.3	SLC13A3-F	22	CTCAAGAGTTTCTTCCCACAGT	77
		SLC13A3-R	19	AGCAGCATGAGAGGAAAGG	
SLC6A20	XM_015102462.1	SLC6A20-F	17	CTGTCCCTGCTGCTCAT	79
		SLC6A20-R	20	GGTCGCTTTCAAATCTGCTC	
SPHKAP	XM_012147712.2	SPHKAP-F	18	CGCTTCTGTCTGCTTTGT	86
		SPHKAP-R	21	GTGAAACACTGACCAACTTCT	
ASZ1	NM_001195309.2	ASZ1-F	22	GCCCTTAAAGAACTGGAAGTAG	85
		ASZ1-R	21	GGAACTCATCACCACTGATTT	
ND6	DQ320083.1	ND6-F	22	AGGGACGTTTATTACTGGTTTA	77
		ND6-R	23	CAATTTCCACCTCCTTATCTTTC	
RARRES2	XM_012143266.2	RARRES2-F	17	GGGCAGTTTGTGAGGCT	91
		RARRES2-R	18	CATTGGGCTTGACCTTGC	
EYA1	XM_012183841.2	EYA1-F	20	CCAATGGCACCGAAGTTAAA	99
		EYA1-R	21	CAATGGCTGAACCTGAGAAAT	
GSN	NM_001246006.1	GSN-F	18	TCATGCTTCTGGACACCT	76
		GSN-R	23	GCTTCTGTCTTCTCTTCTTCTTG	
LAMA2	XM_015097399.1	LAMA2-F	20	ACCTTGAATGCCGATTTGAT	91
		LAMA2-R	23	CCTTGACCGAGTAGTAGTATCTT	
VIM	XM_004014247.3	VIM-F	19	GGAGAGGAGAGCAGGATTT	91
		VIM-R	19	TGTCAACCAGAGGAAGTGA	
**Reference Genes**					
GAPDH[a]	NM_001190390.1	GAPDH-F	23	GGCGTGAACCACGAGAAGTATAA	119
		GAPDH-R	19	CCCTCCACGATGCCAAAGT	
ATP1A1[a]	NM_ 001009360	ATP1A1-F	23	GACTTGAACCGAGGCTTAACAAC	51
		ATP1A1-R	21	TCTGGCTAGGATCTCAGCAGC	
HPRT[a]	NM_001034035	HPRT-F	26	TGGTGGAGATGATCTCTCAACTTTAA	52
		HPRT-R	24	TTCGACAATCAAGACATTCTTTCC	
ACTB	NM_001009784.2	ACTB-F	21	ATGTTTGAGACCTTCAACACC	95
		ACTB-R	17	TCCATCACGATGCCAGT	
TFRC	XM_004003001.2	TFRC-F	20	GAGCTGGACCTGAACTATGA	96
		TFRC-R	22	CAGACCCATATCCCTTATGTCT	

[a] Larruskain et al., 2013
